# Replication Checkpoint: Tuning and Coordination of Replication Forks in S Phase

**DOI:** 10.3390/genes4030388

**Published:** 2013-08-19

**Authors:** Nicole Hustedt, Susan M. Gasser, Kenji Shimada

**Affiliations:** Friedrich Miescher Institute for Biomedical Research, Maulbeerstrasse 66, 4058 Basel, Switzerland; E-Mails: nicole.hustedt@fmi.ch (N.H.); susan.gasser@fmi.ch (S.M.G.)

**Keywords:** checkpoint, replication, Mec1/ATR, Tel1/ATM, kinases

## Abstract

Checkpoints monitor critical cell cycle events such as chromosome duplication and segregation. They are highly conserved mechanisms that prevent progression into the next phase of the cell cycle when cells are unable to accomplish the previous event properly. During S phase, cells also provide a surveillance mechanism called the DNA replication checkpoint, which consists of a conserved kinase cascade that is provoked by insults that block or slow down replication forks. The DNA replication checkpoint is crucial for maintaining genome stability, because replication forks become vulnerable to collapse when they encounter obstacles such as nucleotide adducts, nicks, RNA-DNA hybrids, or stable protein-DNA complexes. These can be exogenously induced or can arise from endogenous cellular activity. Here, we summarize the initiation and transduction of the replication checkpoint as well as its targets, which coordinate cell cycle events and DNA replication fork stability.

## 1. Introduction

Dividing cells go through cycles of cell growth, DNA replication, chromatin condensation, chromosome segregation, and cell division. All these highly complicated processes need to be accurately performed and tightly controlled in order to produce viable offspring. Checkpoints were initially characterized as mechanisms that ensure that a certain process is started only after the previous one has been successfully completed [1]. Originally, yeast genetic studies identified checkpoint genes that ensured cell survival and delayed the onset of mitosis during DNA replication arrest, yet many of these factors are now known to be involved in the DNA damage response [2,3,4]. Thus, in addition to preserving the order of events, checkpoints factors contribute to the maintenance of genome stability [5]. The loss of genome stability has extremely deleterious consequences, including malignant transformation or cell death [6].

Cells are exposed to many types of exogenous and endogenous stress that can modify or damage the DNA. These include oxidative stress, ionizing or UV-light mediated irradiation, and chemical damage, as well as inherent DNA compaction, tightly bound DNA binding proteins, and transcription. Even excessive or premature initiation or replication can cause replicative stress [7], arguing that normal DNA metabolism itself can interfere with DNA replication [8]. This is particularly true in cells with perturbed G1/S control, such as oncogene-transformed cells. In response to excessive DNA damage, wild-type cells arrest the cell cycle in G1 phase, before starting DNA replication, or in G2 phase before entering mitosis [9]. On the other hand, replication fork-associated damage provokes a response that delays progression through S phase, by controlling forks and initiation events [10,11,12].

**Figure 1:**
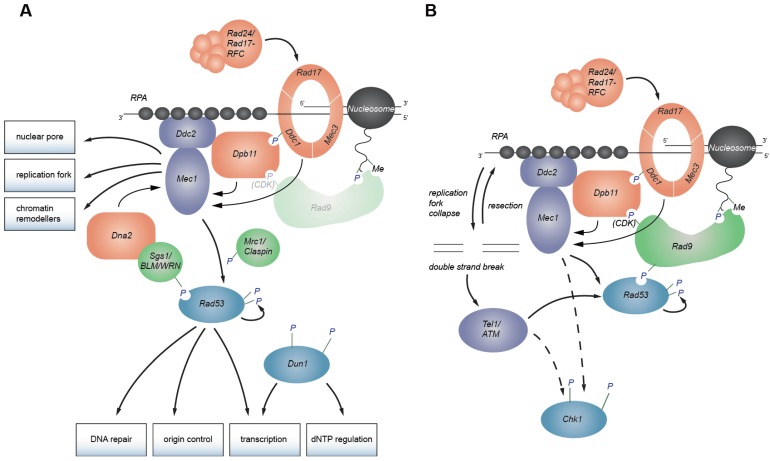
Checkpoint signaling network. (**A**) Replication checkpoint signaling. The yeast equivalent to ATRIP, Ddc2, binds ssDNA that is covered with RPA, while the 9-1-1 checkpoint clamp is loaded onto ds/ssDNA junctions. Dpb11, 9-1-1, and Dna2 (checkpoint sensors, orange) can activate Ddc2-Mec1 (checkpoint kinase, purple). Checkpoint mediators like Mrc1 and Sgs1 (green) help activate Rad53 (checkpoint transducing kinase, blue). Rad53 activates Dun1 and other downstream responses. (**B**) DNA damage checkpoint signaling. Crosstalk between Mec1 and Tel1 (DSB response) can occur, if stalled replication forks collapse, since they can generate DSBs. These are resected to generate ssDNA which activates Mec1. Rad9, the DNA damage checkpoint mediator, can be recruited by histone modifications and also binds, once phosphorylated by CDK, to Dpb11. In addition, both Mec1 and Tel1 can activate the Chk1 kinase.

**Figure 2:**
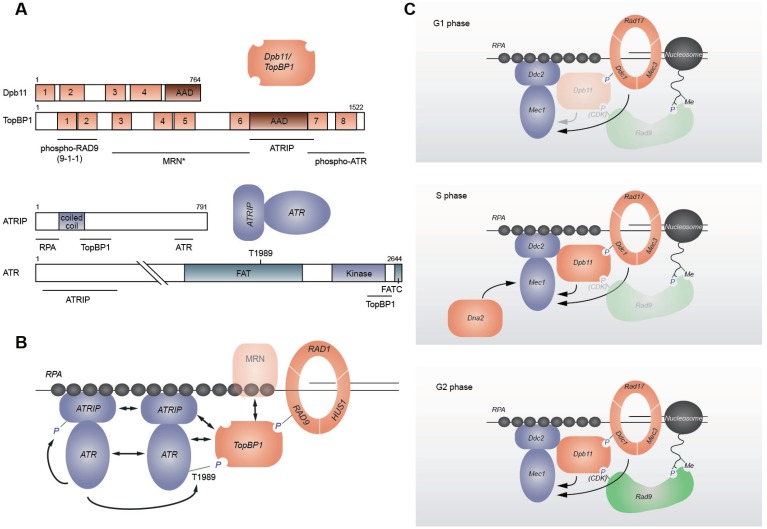
ATR/Mec1 activation. (**A**) Domain architecture of *S. cerevisiae* Dpb11, human TopBP1^Dpb11^, human ATRIP^Ddc2^, and human ATR^Mec1^. Numbered brown boxes indicate BRCA1 C-terminal (BRCT) domains. Underlined regions interact with indicated proteins. * MRN^MRX^ interaction shown for *Xenopus* TopBP1^Dpb11^. (**B**) Mammalian ATR^Mec1^ activation. TopBP1^Dpb11^ is recruited by RAD9^Ddc1^ phosphorylation and interacts with ATRIP^Ddc2^ and ATR^Mec1^. *Xenopus* TopBP1^Dpb11^ may be recruited through MRN^MRX^. ATR^Mec1^ autophosphorylates, and this may also contribute to interaction with TopBP1^Dpb11^. ATRIP^Ddc2^ and ATR^Mec1^ form higher-order oligormers. (**C**) Cell cycle specific *S. cerevisiae* Mec1 activation. In G1 phase Ddc1, a subunit of the 9-1-1 checkpoint clamp, is the predominant Mec1 activator. In S phase, Ddc1, Dpb11, and Dna2 are able to activate Mec1. In G2 phase, both Ddc1 and Dpb11 can activate Mec1. Dpb11 is recruited through phosphorylated Ddc1 and CDK-mediated phosphorylation of Rad9, which in turn binds to modified histones. AAD—ATR/Mec1 activation domain; FAT—FRAPP, ATM, TRRAP domain; kinase—kinase domain; FATC—FAT C-terminal domain.

At the heart of all DNA damage checkpoint responses, are the two kinases Tel1 (telomere maintenance 1; ATM in mammals, see Table 1 for an overview of checkpoint protein names in several model organisms) and Mec1 (Mitosis Entry Checkpoint 1; ATR in mammals) (Figure 1) [3]. Both are phosphoinositide 3-kinase (PI3K)-related kinases (PIKKs) that share significant sequence homology and phosphorylate an overlapping set of substrates. Both show a preference for serine or threonine residues followed by glutamine ([S/T]Q) or a hydrophobic residue [13,14,15,16]. Often these target sites are found in SQ/TQ cluster domains (SCDs) [17]. All PIKKs, not only Mec1 and Tel1, share a common domain architecture in which the kinase domain is flanked by both FRAPP, ATM, TRRAP (FAT), and FAT C-terminal (FATC) domains (Figure 2A), all being conserved alpha-helical regions [18,19]. Because FAT and FATC domains are always present in combination, it has been suggested that these two domains interact with each other, potentially providing a scaffold or binding sites for other proteins [19].

In budding yeast, Mec1 is active even in an unperturbed S phase, as it can regulate dNTP levels and replication initiation without blocking cell cycle progression [20,21]. ATR^Mec1^ becomes hyperactivated in response to a wide variety of DNA insults and is essential for cell viability, whereas ATM^Tel1^ is activated primarily by double-strand breaks (DSBs) and its loss in budding yeast is not lethal. Nonetheless, in mammalian cells, mutation of either homolog leads to an elevated predisposition towards cancer [18]. Once localized to the site of DNA damage and activated by DNA damage sensing proteins, either kinase can initiate a signaling cascade that transduces the signal through mediator proteins Mrc1 and Rad9 (Claspin, BRCA1, MDC1 and 53BP1 in mammals) to the effector kinases Rad53 and Chk1 (CHK2 and CHK1 in mammals) (Figure 1) [22,23,24,25]. Effector kinases are transiently recruited to sites of DNA damage and are released after their activation [26,27], allowing transmission of the checkpoint response to a range of effector proteins [28]. In addition to the effector kinases, Mec1 and Tel1 also phosphorylate proteins bound at sites of damage, such as budding yeast histone H2A (the H2AX variant in mammals), generating γH2AX, to provoke local chromatin changes [29].

**Table 1:** Conserved checkpoint proteins and their functions.

*S. cerevisiae*	*S. pombe*	*H. sapiens*	*Function*
Rad24-RFC	Rad17-RFC	RAD17-RFC	RFC-like complex, 9-1-1 clamp loader
Ddc1-Rad17-Mec3	Rad9-Rad1-Hus1	RAD9-RAD1-HUS1	9-1-1 complex, DNA damage checkpoint clamp, Mec1 activation
Dpb11	Cut5/Rad4	TOPBP1	Mec1 ATR activation
Dna2	Dna2	DNA2	Mec1 activation in S phase
Mre11-Rad50-Xrs2	Mre11/Rad32-Rad50-Nbs1	MRE11-RAD50-NBS1	MRX/MRN complex, DSB resection, Tel1/ATM recruitment
Mec1-Ddc2	Rad3-Rad26	ATR-ATRIP	checkpoint signaling kinase
Tel1	Tel1	ATM	checkpoint signaling kinase
Mrc1	Mrc1	Claspin	fork-associated, checkpoint mediator
Rad9	Crb2	53BP1, BRCA1	checkpoint mediator
Sgs1	Rqh1	BLM, WRN	fork-associated, Rad53 activation
Rad53	Cds1	CHK2	effector kinase
Chk1	Chk1	CHK1	effector kinase

**Gene name abbreviations:** Rad24 (radiation sensitive 24), RFC (replication factor c), Ddc1 (DNA damage checkpoint 1), Mec3 (Mitosis entry checkpoint 3), Hus1 (hydroxyurea sensitive 1), Dpb11 (DNA polymerase B 11), Cut5 (cell untimely torn 5), TOPBP1 (DNA topoisomerase 2 binding protein 1), Dna2 (DNA synthesis defective 2), Mre11(meiotic recombination 11), Xrs2 (X-ray sensitive 2), Nbs1 (Nijmegen breakage syndrome 1), ATR (ATM and Rad3-related), ATRIP (ATR interacting protein), Tel1 (telomere maintenance 1), ATM (Ataxia telangiectasia mutated), Mrc1 (mediator of the replication checkpoint 1), Crb2 (cut5 repeat binding 2), 53BP1 (tumor suppressor p53 binding protein 1), BRCA1 (breast cancer 1, early-onset), Sgs1 (slow growth suppressor 1), rqh1 (RecQ-type DNA helicase 1), BLM (Bloom syndrome protein), WRN (Werner syndrome ATP-dependent helicase), Cds1 (checking DNA synthesis 1), CHK2 (checkpoint kinase 2), CHK1 (checkpoint kinase 1).

DNA damage occurs in all stages of the cell cycle, yet cells are particularly vulnerable to insults during DNA replication, when the double helix is unwound. Indeed, in S phase, defects in one strand can have serious consequences on the integrity of the daughter chromosome. Moreover, the single-stranded DNA (ssDNA) that is generated during replication, is intrinsically more labile than double-stranded (dsDNA) [30]. Consistently, sites that slow the DNA replication fork have been shown to correlate with sites of enhanced genome fragility [31]. To cope with this danger, cells provide a surveillance mechanism called intra-S-phase or DNA replication checkpoint (Figure 1A). This checkpoint slows genome replication by inhibiting the firing of late origins [10,11], and protects stalled replication forks by preventing their conversion to DSBs and/or reducing recombination events [32,33,34]. Consistently, it has been shown that the loss of replication checkpoint factors provokes high levels of spontaneous gross chromosomal rearrangements in budding yeast [35]. The factors involved in this checkpoint are highly conserved and many, including ATR itself, have tumor suppressor roles in mammals [8].

Here we review recent findings on the replication checkpoint. We will first discuss the nature of the DNA lesions that provoke a checkpoint response. We then describe the mechanism of ATR^Mec1^ activation and summarize the functions served by the replication checkpoint, especially with respect to replication fork stability. We will discuss how cells downregulate the checkpoint signal to resume the cell cycle after the insult has been removed, and finally examine the coordination between two checkpoint PIKK kinases, ATR^Mec1^ and ATM^Tel1^. Although we focus primarily on insights from studies in budding yeast, we relate those findings to results obtained from other organisms.

## 2. Replication Checkpoint Initiation

### 2.1. Lesions that Activate the Checkpoint

Replication forks themselves play a critical role in inducing a checkpoint signal. Only when a critical number of replication forks initiate and encounter lesions, will the replication checkpoint signal become robust [34,36]. This has seeded the notion of a threshold for activation of the replication checkpoint. After treatment with a replication stress-inducing drug (hydroxyurea, HU), long stretches of ssDNA (about 200 nucleotides) are exposed at stalled forks [33]. These extended stretches of ssDNA themselves contribute to the induction of the checkpoint response, but they are not sufficient: a double-stranded primer with a free 5' end is also required [37]. The ds-ssDNA junction structure can arise from a variety of replication and repair processes, such as lagging strand DNA synthesis, nucleotide excision repair [38], or from resection at DSBs. This structure is recognized by the 9-1-1 checkpoint clamp and its loading factor (see below and Figure 1A,B). At DSBs ATM^Tel1^ is recruited and activated initially by the Mre11-Rad50-Nbs1^Xrs2^ complex, which then promotes resection. Resection generates ssDNA and a ds-ssDNA junction, which in turn activate ATR^Mec1^ [39,40,41,42] (see also Section 6). Both at resected DSBs and at stalled replication forks, ssDNA is rapidly coated with the trimeric ssDNA binding complex RPA (replication protein A) [43]. RPA-bound ssDNA interacts with ATRIP^Ddc2^, an essential cofactor of the ATR^Mec1^ kinase [44,45] (Figure 1). Mutations in RPA that disrupt its interaction with ATRIP^Ddc2^ reduce checkpoint activation [44,46,47,48]. In budding yeast, the RPA-Ddc2 interaction also requires Mec1, suggesting either that there may be an independent RPA binding surface on Mec1, or that Mec1 changes Ddc2 conformation in a way that favors RPA interaction [49].

The ds-ssDNA junctions are recognized by the Rad24-RFC complex that loads the 9-1-1 checkpoint clamp (Ddc1, Rad17 and Mec3 in *S. cerevisiae*). *In vitro* analysis argues that 9-1-1 can be loaded at both 3' and 5' junctions, although if RPA is bound to the ssDNA, the 9-1-1 complex prefers to load at 5' junctions. These structures, together with RPA, are sufficient to activate the ATR^Mec1^ checkpoint in a cell-free system [37,50]. In budding yeast, Mec1 phosphorylates the Ddc1 subunit (human RAD9) of the 9-1-1 complex, which can then recruit Dpb11 (human TopBP1). TopBP1^Dpb11^ further stimulates ATR^Mec1^ kinase activity [51,52,53,54,55,56,57]. This indicates that in addition to its loading onto ssDNA, ATR^Mec1^-ATRIP^Ddc2^ needs to contact an activator in order to induce the checkpoint response (see Section 2.3). In addition to this, several studies have suggested that mismatch repair factors at the site of DNA damage provide an alternative means to recruit and activate ATR^Mec1^ [58,59,60].

### 2.2. Drugs Used to Induce and Study Checkpoint Responses

**Figure 3:**
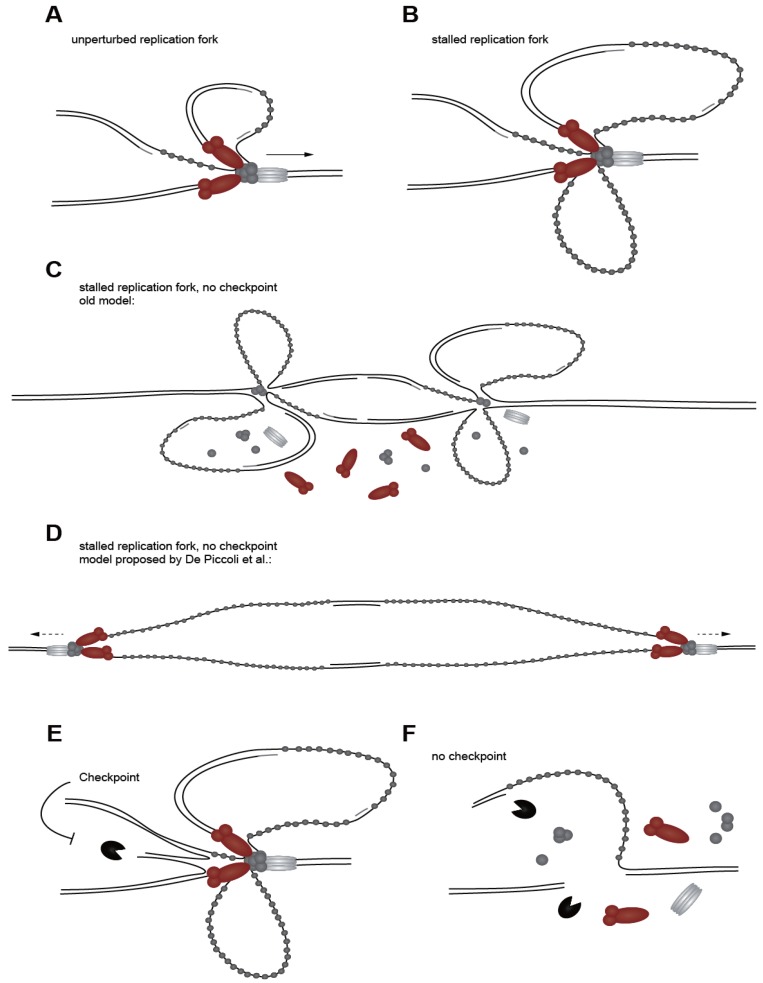
Replication fork stabilization. (**A**) A normal replication fork with leading and lagging strand polymerases, replicative helicase, and short ssDNA stretches coated with RPA. (**B**) When replication forks stall and helicase and polymerases become functionally uncoupled, long ssDNA stretches are exposed that lead to checkpoint activation. (**C**) ChIP experiments have indicated that replisome factors are lost from stalled replication forks if the replication checkpoint is not functional. (**D**) New data suggest that replisomes stay intact, but move away from replication origins without incorporating nucleotides. This leads to long ssDNA stretches. (**E**) The checkpoint regulates nucleases that may target structures (e.g., reverse forks) that arise at stalled replication forks. (**F**) If nuclease regulation fails due to checkpoint dysfunction, this may lead to uncontrolled processing and could result in double-strand breaks.

As mentioned above, special DNA structures initiate the ATR^Mec1^ checkpoint response. To study the replication checkpoint and downstream responses *in vivo*, a variety of DNA-damaging and fork stalling agents are used, and these provoke the checkpoint response in different ways (summarized in Table 2). Natural replication fork stalling can also occur, generally due to secondary DNA structures (e.g., G-quadruplexes), RNA-DNA hybrids found at genes, tightly bound transcription complexes (e.g., at rDNA or tRNA genes) or specific protein-DNA complexes, like that formed by the replication fork barrier protein (Fob1), which prevents forks from colliding with RNA PolI in the rDNA repeats [61,62]. Natural fork pausing, however, does not provoke a global checkpoint response. We note that the proteins involved in a chemically induced checkpoint responses depend on the type of damage induced and can vary with the dose applied, as demonstrated recently for camptothecin [63]. Further complexity in the checkpoint network stems from differences in cell type or genetic background. For example, the checkpoint response is more prone to be activated in cells deficient for DNA repair, such the *rad18* mutant, which is deficient for post-replication repair [64,65]. Other lesions that expose primed ssDNA and activate the replication checkpoint arise when replicative helicase and polymerase functions are uncoupled [66] (Figure 3A,B). Here we summarize commonly used treatments that induce the checkpoint response, and highlight differences in the responses they elicit.

**Table 2:** *S. cerevisiae* checkpoint responses differ, depending on the treatment.

Treatment/Impediments	Mode of action	Result	Responders ( *S.cerevisiae*)
**hydroxyurea (HU)**	inhibits ribonucleotide reductase—dNTP pools become depleted	uncoupling of helicase and polymerase function; ssDNA is exposed	Mec1, Mrc1, Sgs1
**aphidicolin**	inhibits DNA polymerases	uncoupling of helicase and polymerase function; ssDNA is exposed	Mec1, Mrc1
**methylmethanesulfonate (MMS)**	alkylates DNA	uncoupling of helicase and polymerase function; ssDNA is exposed; in addition DNA repair takes place, that also leads to ssDNA; requires replication forks to induce checkpoint response	Mec1, Rad9 (Mrc1, Sgs1)
**ultraviolet light/4-NQO**	induces Thymidine dimerization	induces DNA repair, that leads to ssDNA	Mec1, Rad9 Mrc1
**crosslinking agents (cisplatin/nitrogen mustard)**	causes DNA inter-strand crosslinks	both helicase and polymerase are blocked; in addition DNA repair takes place, that also leads to ssDNA	Mec1/Tel1, Rad9
**ionizing irradiation (IR)/bleomycin**	causes single and double strand breaks	breaks are directly recognized by MRX-Tel1; resection leads to ssDNA	Mec1/Tel1; Rad9
**camptothecin (CPT)**	inhibits Topoisomerase I, keeps it in a DNA-bound confirmation	both helicase and polymerase are blocked; double strand breaks are actively induced by DNA repair machinery	Mec1/Tel1, Rad9
**natural fork barriers (rDNA, t-RNA genes, transcription**	slow down replisome progression	both helicase and polymerase are slowed down	-

One commonly used means to trigger the replication checkpoint is to treat cells with either hydroxyurea (HU) or aphidicolin. HU inhibits ribonucleotide reductase (RNR) by reducing the reactive tyrosyl radical in the active center of the enzyme [67]. When replication is initiated, dNTP pools are rapidly depleted if RNR is inhibited [68], and this leads to a stalling of DNA polymerases. Aphidicolin, on the other hand, directly inhibits DNA polymerases [69] without affecting the replicative helicase [70]. Accordingly, polymerases are blocked, but MCM helicases continue to move, generating long stretches of ssDNA that trigger the replication checkpoint [37].

Interstrand crosslinks (ICs), such as those caused by cisplatin, do the opposite: they tend to block the MCM helicase in front of the replication fork. There is no uncoupling of helicase and polymerase, and hence no immediate activation of the replication checkpoint. However, often the replication forks pause 20–40 nucleotides before reaching the IC lesion, and often structure-specific nucleases will process the template or nascent strand, generating ssDNA [71,72], which in turn leads to checkpoint activation.

Methyl methanesulfonate (MMS) creates bulky lesions by alkylating DNA. Alkylation alone does not elicit the checkpoint response, but requires that the replication fork collides with the DNA adduct. Therefore, MMS-induced checkpoint responses are restricted to S-phase cells [34]. Although both MMS and HU activate an S-phase checkpoint, it is important to note that they do not provoke equivalent responses [24,46,73,74]. In response to MMS, cells activate several repair pathways, including base excision repair (BER), DNA damage tolerance pathways such as trans-lesion synthesis [75], and homologous recombination (HR). However, since DSBs are not detected on MMS, it is not clear whether it is the repair process itself, or an uncoupling of leading and lagging strand synthesis, which provokes what appears to be a combined replication/DNA damage checkpoint response [76].

Ionizing radiation (IR) or treatment with bleomycin or its derivatives (e.g., Zeocin^®^) cause DSBs, which activate the DNA damage response initially through the ATM^Tel1^ kinase, and after processing, through ATR^Mec1^ [77]. It should be kept in mind that IR or bleomycin derivatives induce oxidative damage and single-strand nicks much more efficiently than DSBs. At sufficiently high doses, UV light also provokes an ATR^Mec1^-dependent response [39], for the pyrimidine dimers caused by UV treatment are recognized by the nucleotide excision repair (NER) machinery, which itself creates a ssDNA patch during the repair process [38]. While these lead to ATR^Mec1^ activation, the pathway involves Rad9 (homologue of 53BP1 or BRCA1), a mediator typically implicated at DSBs [78]. In mammals, Mre11-Rad50-Nbs1^Xrs2^ (MRN) nuclease has been shown to be involved in UV-dependent ATR^Mec1^ activation, possibly again by creating a single-strand patch [79]. 

Camptothecin inhibits topoisomerase I (Top1) by blocking religation after the enzyme has made a ssDNA nick and becomes covalently linked to the DNA end [80]. These structures arrest replication forks and are cytotoxic [81], yet they only provoke a mild checkpoint response [82], presumably because the lesion is not accompanied by helicase-polymerase uncoupling nor extensive resection. The so-called replication run-off model [83] has indicated that replication forks running into these ssDNA nicks are converted into DSBs, which generally depend on a recombination-dependent mechanism for replication fork restart [63,84]. Recent data indicate that torsional stress generated by Top1 inhibition may lead to fork slowing, and suggest that the formation of a DSB is an active process involving cleavage by the endonuclease Mus81. An artificial system that generates a similar lesion uses a mutant form of the site-specific Flp recombinase to generate a covalent protein-DNA complex, adjacent to a ssDNA nick [85]. This lesion, like the Top1-camptothecin complex, does not induce a checkpoint response in wild-type yeast, but recruits the recombination machinery for repair following collision with a replication fork (L. Bjergbaek, personal communication). This illustrates the broad range of responses elicited by exogenous agents, and underscores the importance of highlighting the type of damaging agent used.

### 2.3. Mec1/ATR Activation

An accumulation of RPA-coated ssDNA recruits ATR^Mec1^-ATRIP^Ddc2^, just like a DSB bound by MRN^MRX^ recruits ATM^Tel1^. However, whereas MRN^MRX^ also activates ATM^Tel1^, ssDNA-RPA is not sufficient to induce ATR^Mec1^ activation. As discussed above, ds-ssDNA junctions that recruit the 9-1-1 checkpoint clamp are also required for activation (see Section 2.1). In budding yeast, Ddc1, a subunit of the 9-1-1 complex that binds the ds-ssDNA junction, has been shown to be capable of activating Mec1 alone under low salt conditions *in vitro* [86,87], just as the artificial juxtaposition of multiple Ddc1 and Ddc2 molecules can activate Mec1 *in vivo* [88]. In higher organisms, on the other hand, RAD9^Ddc1^ instead creates a binding site for the ATR^Mec1^ activator TopBP1^Dpb11^ [51,52,53]. TopBP1^Dpb11^ contains eight BRCA1 C-terminal (BRCT) domains (Figure 2A) and interacts with phosphorylated RAD9^Ddc1^ through its BRCT domains 1 and 2. In addition, the MRN^MRX^ complex has been shown to recruit TopBP1^Dpb11^ through its BRCT domains 3-6 (Figure 2B) [89]. Overexpression of a domain of TopBP1^Dpb11^ that sits between its BRCT motifs 6 and 7 (called AAD for ATR activation domain), also leads to ATR^Mec1^ activation. Indeed, one can bypass the need for the intact 9-1-1 clamp by tethering the AAD to PCNA or histone H2B [51,90]. TopBP1^Dpb11^ itself binds to ATRIP^Ddc2^, and mutations within its TopBP1^Dpb11^ binding region can block ATR^Mec1^ activation. Finally, a region of ATR^Mec1^, between the kinase and the FATC domain, is important for TopBP1^Dpb11^-mediated ATR^Mec1^ activation [54] (Figure 2A,B). Here, however, molecular details are scarce, as there are no structural data available for ATR^Mec1^. This RAD9^Ddc1^-TopBP1^Dpb11^ pathway for ATR^Mec1^ activation is also found in budding yeast. Dpb11 is recruited by Ddc1, which is phosphorylated by Mec1 [56], although either Ddc1 or Dpb11 can activate Mec1 on its own [55,91,92]. The responsible regions of Dpb11 and Ddc1 have been mapped to their unstructured C-terminal tails, within which two conserved hydrophobic residues are important for Mec1 activation [87,92,93].

How Ddc1 and Dpb11 act together to activate Mec1 is still under debate, and it may vary in a cell-cycle dependent manner (Figure 2C). Burgers’ laboratory suggests that whereas the 9-1-1 subunit Ddc1 is responsible for the activation of Mec1 in response to DNA damage in G1 phase, 9-1-1 and Dpb11 cooperate to activate Mec1 in G2/M phase [92,94]. Dpb11 interacts with phosphorylated Rad9, which is modified by a cell-cycle regulated Cyclin-dependent kinase (CDK). Since CDK is not active in G1, this could explain why Dpb11 function is cell-cycle specific [93]. In contrast, Puddu *et al.* have shown that Dpb11 and 9-1-1 act together in G1, while 9-1-1 is the predominant Mec1 activator in G2 [95]. Fission yeast Rad4/Cut5^Dpb11^ similarly assists Rad3^Mec1^ activation in G1, when DSB resection is restricted [96]. This observation suggests that Rad4/Cut5^Dpb11^ compensates for limited ssDNA to promote full Rad3^Mec1^ activation in G1 phase [96].

In S-phase cells, several proteins have been reported to activate Mec1, apparently in a redundant manner. The 9-1-1 complex is recruited to stalled replication forks, and facilitates Rad53 phosphorylation [27,97]. However, *dpb11* or *ddc1* mutations that interfere with Mec1 activation (or a mutation defective in 9-1-1 loading such as *rad24*Δ), alone or in combination, show only mild defects in Rad53 phosphorylation in response to replication stress [87,92,98]. Recently, Burgers’ laboratory has reported that Dna2, a conserved nuclease-helicase that is essential for Okazaki fragment maturation, has a role in Mec1 activation in S phase. *TEL1* and *DDC1* deletions (which also compromise Dpb11-mediated Mec1 activation) were combined with a mutation in the Mec1 activation domain of Dna2, and this eliminated Rad53 activation upon HU treatment in S phase [99]. These data indicate that Dna2 functions as a third factor contributing to Mec1 activation in S phase. Here it is important to note that Dna2 binds the yeast RecQ helicase Sgs1, and that the two factors co-activate each other [100]. Sgs1 also promotes replication stress-dependent checkpoint activation, and the checkpoint defects of a *SGS1* deletion are strongly aggravated by mutations in 9-1-1 or the *RAD24* gene [97,101,102]. Sgs1 directly binds Rad53 in a Mec1-dependent manner, arguing that it does not simply generate structures that activate and require Mec1 [103].

Whereas Ddc1, Dpb11 and Dna2 were all shown to enhance Mec1 catalytic activity *in vitro,* the molecular details of how they act on Mec1 are unclear. Rather than stimulating Mec1 through its kinase domain, they may serve as scaffolds that bring factors closely together. Indeed, Berens and Toczyski have shown that an artificial co-localization of Ddc2 and the replication checkpoint mediator Mrc1 elicits a downstream Rad53 kinase response in the absence of Dpb11 and Ddc1 [98]. It remains possible the Dna2 serves as the crucial activator in this case, and it would be interesting to see how a *dna*2 mutant defective for Mec1 activation, or the *dna2* *dpb11* and *dna2 ddc1* double mutants, would behave in the Toczyski assay.

Another level of ATR^Mec1^ regulation may be inherent to the kinase itself. Recently it has been reported that human ATR^Mec1^ can autophosphorylate in *trans*, and that this phosphorylation correlates with ATR^Mec1^ activation [104,105]. It has been suggested that ATR^Mec1^ autophosphorylation assists its binding to TopBP1^Dpb11^, which further activates the kinase [104] (Figure 2B). However, another study concluded that mutation of the same autophosphorylation site does not have a strong impact on ATR^Mec1^ function [105], and the relevant target residue, Thr 1989, is not conserved in budding and fission yeast [105]. Indeed, a study that mutagenized all [S/T]Q sites in checkpoint proteins in fission yeast did not find a single [S/T]Q residue in Rad3^Mec1^ critical for its function [106]. However, since the ATR^Mec1^ autophosphorylation site does not match the [S/T]Q consensus [104,105], these results are inconclusive. It remains to be seen whether a similar autophosphorylation mechanism exists for Rad3^Mec1^ in fission yeast or for Mec1 in budding yeast.

ATR^Mec1^ may be also controlled by regulated protein complex formation. It has recently been determined that Nek1 (Never in mitosis A-related kinase 1) promotes ATR^Mec1^-ATRIP^Ddc2^ association in a DNA damage independent manner. The responsible phosphorylation site is unknown, but Nek1 does not seem to target ATR^Mec1^ Thr 1989 directly [107]. Functional complexes may require disruption of dimers. ATM^Tel1^ forms inactive dimers, which dissociate upon autophosphorylation after DSB induction [108]. ATR^Mec1^-ATRIP^Ddc2^ can also form oligomers [109,110,111,112,113] and it has been speculated that the oligomerization of ATR^Mec1^-ATRIP^Ddc2^ regulates kinase activity, even though oligomerization is independent of DNA damage or replication stress [110,112]. There are conflicting reports on the size of ATR^Mec1^-ATRIP^Ddc2^ complexes, ranging from 300 to 1,000 kD in size [86,110,114]. In humans, both the coiled-coil domain of ATRIP^Ddc2^ and ATR^Mec1^ contribute to oligomerization [109] (Figure 2A). Mutation of the human ATRIP^Ddc2^ coiled-coil domain does not impair chromatin binding, but impairs foci formation and signaling. Interestingly, this coiled-coil mutation shows stronger defects in the replication checkpoint than in the G2/M damage checkpoint [109,110]. In contrast, it has been reported that in *Xenopus* the ATRIP^Ddc2^ coiled-coil domain is dispensable for both oligomerization and CHK1^Chk1^ phosphorylation [114], indicating species or cell-type specific differences.

## 3. Activation of Effector Kinases

Once ATR^Mec1^ and ATM^Tel1^ have been activated, these kinases signal to the downstream effector kinases, Rad53 and Chk1 (Figure 1). Although Rad53 is more closely related to CHK2^Rad53^ by sequence, its function is taken over by CHK1^Chk1^ in higher organisms. In response to stalled replication forks, the signaling of the replication checkpoint is mediated primarily through Rad53 in budding yeast, or by its functional homolog CHK1^Chk1^ in mammalian cells. Rad53 contains a kinase domain which is flanked by two Forkhead associated (FHA) domains, that can bind phosphorylated proteins [115]. Mutations of critical residues in the FHA domains have revealed that full Rad53 activation requires at least one functional FHA domain, and that mutations of FHA2 show slightly stronger defects [16,116,117]. N-terminal of each FHA domain is an [S/T]Q cluster domain [118], which becomes modified at multiple residues by either Mec1 or Tel1 [23,24,119]. Nonetheless, genetic evidence indicates that either Mec1 or Tel1 is required, but not sufficient, for Rad53 activation [73,120]. Rad53 activation is facilitated by at least two mediator proteins: the budding yeast Rad9 fulfills this role in the DNA damage checkpoint, while Mrc1 serves as mediator during replication checkpoint activation [16,25,120,121]. Interestingly, Rad53 also appears to be phosphorylated in a cell-cycle dependent manner, and this phosphorylation may fine-tune the checkpoint response [122]. Here we will first summarize the well-characterized molecular mechanism of Rad53 activation by Rad9, and then review current knowledge about Mrc1.

### 3.1. Rad53 Activation Is Mediated by Rad9 in Response to DNA Damage

Rad9 was the first cell-cycle checkpoint protein identified in budding yeast, and it has a key role as an adaptor for activating Rad53 in the DNA damage response, yet it has little or no role in replication checkpoint triggered by HU-arrested forks [2,123]. Rad9 does not possess enzymatic activity, but contains both tandem Tudor and BRCA1 C-terminal (BRCT) domains. Thus, the mammalian proteins BRCA1, MDC1 and 53BP1 are all considered to be functional homologs of Rad9. The Rad9 tandem Tudor domains can bind to histone H3 methylated on lysine 79 [124], which is deposited throughout the genome by the methyltransferase Dot1 [125]. In addition, Rad9 binds histone H2A phosphorylated on serine 129, using its tandem BRCT domains [126]. The H2A phosphorylation is mediated by both Tel1 and Mec1 at sites of DNA damage [29,32,127], and both H3K79 methylation and phosphorylated H2A are thought to recruit Rad9 to damaged sites. Consistently, strains bearing *dot1*Δ or phospho-acceptor mutations in H2A show defects in the G1 checkpoint activation [124,126,128,129]. The G2 checkpoint, on the other hand, still functions in the *dot1* mutant. Because the G2 checkpoint activity is lost in a *dpb11 dot1* double mutant, it appears that Dpb11 may also recruit Rad9 in G2 (see Section 2.3) [56,93].

Rad9 becomes phosphorylated by Mec1/Tel1 in response to DNA damage, and is required for efficient Rad53 activation [73]. It has been shown that phosphorylated Rad9 can bind to the Rad53 FHA domains, with a preference for FHA2 [16,73,117,130,131]. This, together with the observation that the autophosphorylation of Rad53 is concentration-dependent, has led to the hypothesis that phosphorylated Rad9 locally increases Rad53 concentration, providing a scaffold for efficient Rad53 autophosphorylation and activation [26,132]. In this model, Mec1 would be only required for initial Rad9 phosphorylation, and might not necessarily act directly on Rad53. However, more recent studies have shown that direct phosphorylation of Rad53 by Mec1/Tel1, and not only of Rad9, is required for Rad53 activation [16,132]. Indeed, the mutation of Mec1/Tel1 target sites in Rad53’s N-terminal [S/T]Q cluster domain reduced viability, replication and damage checkpoint functions, as well as its kinase activity [118].

Activation of the mammalian Rad53 homolog CHK2^Rad53^ or *S. pombe* Cds1^Rad53^ requires phosphorylation of one specific residue by upstream kinases (threonine 68 or 11, respectively) [133,134]. In the case of Rad53, the [S/T]Q sites in the N-terminal SCD seem to be redundant, and phosphorylation at multiple sites is important for activation [16,118]. Once Rad53 has been primed by Mec1/Tel1 and fully activated through autophosphorylation, Rad9 seems to release Rad53, enabling the transduction of the checkpoint responses throughout the nucleus [26]. Mec1/Tel1 phosphorylation of Rad9 also leads to oligomerization of Rad9. The oligomerization may be dispensable for Rad53 activation, whereas it is needed for maintenance of the checkpoint [135]. Intriguingly, phosphorylation of Rad9 by Rad53 disrupts Rad9 oligomerization, providing a negative feedback mechanism for checkpoint regulation [135]. 

### 3.2. Mrc1 Serves as a Mediator in the Replication Checkpoint

Whereas Rad9 acts in response to DNA damage in G1 and G2, Mrc1 is a key mediator protein for Rad53 activation in the context of DNA replication [25,136,137]. Mrc1 is a component of the replisome, and it travels along with replication forks [27,136]. Mrc1 enhances Rad53 activation during replication stress, but does not activate Mec1 kinase activity per se. Rather, it seems to positively influence the enzyme-substrate interaction between Mec1 and Rad53, and could, therefore, recruit Rad53 to stalled forks to facilitate Rad53-Mec1 interaction [138]. Analogously, Claspin, the Mrc1 homolog in higher eukaryotes, contributes to replication checkpoint activation by interacting with CHK1^Chk1^, a functional homolog of Rad53, and facilitating its activation [139,140,141].

In addition to its checkpoint function, Mrc1 appears to have a structural role in replication fork maintenance, as it binds to the replisome via the Csm3-Tof1 fork protection complex [142]. Mrc1 interacts with Pol ε and Mcm6 [143,144], and Tof1 and Csm3 interact with Mcm2 [142]. Thus, it has been suggested that this complex forms a bridge between the leading strand polymerase ε and the replicative helicase [143]. Consistently, replisome structure is aberrant in the *mrc1*Δ mutant; *mrc1*Δ cells proceed faster through S phase, show an uncoupling of the replisome from the site of DNA synthesis and have impaired recovery from HU arrest [25,27,145,146]. Mrc1 becomes phosphorylated by Mec1 and a mutant in which all Mrc1 [S/T]Q sites are mutated to AQ, shows a defect only in checkpoint signaling, but not in replisome progression [25,136]. By using this *mrc1*-AQ mutant, an alternative function for Mrc1 in the replication checkpoint has been suggested, namely, phosphorylation of Mrc1 may stabilize the association of Mec1 with sites of stalled replication forks, thereby creating a positive feedback for Mec1 function [147]. Although Mrc1 activates the replication checkpoint in response to replication stress, loss of it can be compensated by Rad9 [25]. Indeed, Rad9 accumulates at stalled replication forks in *mrc1*Δ cells on HU [27]. It is therefore likely that loss of Mrc1 checkpoint function creates DSBs or damage structures that provoke Rad9-dependent Rad53 activation. This is a much more likely option than that Mrc1 and Rad9 are equivalent in their mode of action, given that Mrc1 is part of the replisome, and Rad9 clearly is not.

### 3.3. A Role for Sgs1 in Rad53 Activation

Another factor that has been shown to be involved in replication checkpoint signaling and which helps activate Rad53, is the budding yeast RecQ helicase, Sgs1 [97,103]. Sgs1 interacts with Dna2, RPA and Rad53, and is constitutively associated with replication forks [100,103,148]. The deletion of *sgs1* alone destabilizes DNA polymerases α and ε when replication forks are stalled by HU [148], and this effect is far more pronounced when combined with either *mrc1*Δ or the S-phase specific mutant allele of *MEC1*, *mec1-100* [32,112,148,149,150]. This leads to a synergistic arrest of growth and failed fork recovery in response to HU, and a loss of dNTPs incorporation, as both polymerases α and ε are lost from the replisome [148]. One explanation of the observed synergy may be that *mec1*-*100* generates fold back structures that need Sgs1 for resolution. However, Sgs1 also participates in the activation of Rad53, particularly in response to HU arrest [97]. Sgs1 itself contains a [S/TQ] cluster that is phosphorylated in a Mec1-dependent manner *in vivo* and *in vitro* [103]. When phosphorylated, this domain of Sgs1 binds Rad53, again both *in vivo* and *in vitro* [97,103]. Therefore, Sgs1 serves as a replication checkpoint mediator that recruits Rad53 to stalled forks, acting in much the same way as Rad9 acts at DSBs. Mrc1 and Sgs1 have been found to be epistatic for their function in Rad53 phosphorylation, although Sgs1 functions in parallel with Rad24 and 9-1-1, and the double mutants are highly compromised for the activation of Rad53 at stalled forked [97,102]. A RecQ homologue in mammals, the WRN^Sgs1^ helicase, has also been shown to facilitate the ATR^Mec1^-CHK1^Chk1^ checkpoint pathway in response to camptothecin [151], indicating that RecQ function in checkpoint signaling may be conserved.

## 4. Targets of the Replication Checkpoint

An activated replication checkpoint cascade transduces a multitude of stimuli that control cell-cycle and replication-fork recovery. Here, we summarize the downstream pathways that are influenced by the checkpoint. We focus on relevant replication fork targets at the end of this section, to the extent that they are known.

### 4.1. Cell Cycle Regulation

In all eukaryotic species, checkpoint effector kinases play a central role in cell-cycle arrest upon checkpoint activation, even though the mechanism of arrest differs. In fission yeasts and higher eukaryotes, CHK1^Chk1^ and CHK2^Rad53^ negatively regulate CDC25^Mih1^ phosphatases that remove inhibitory phosphorylation on cyclin-dependent kinase (CDK) [152,153,154,155]. Checkpoint-dependent phosphorylation of CDC25^Mih1^ down-regulates its activity through inhibition of its nuclear localization by binding 14-3-3, a nuclear-cytoplasmic shuttling protein, and through degradation by the SCF^ΒTrcp^ ubiquitin ligase [156,157,158]. Therefore, DNA replication and the damage checkpoint down-regulates the CDK cell cycle engine, thereby blocking G2/M transition. In *S. cerevisiae*, however, this cell-cycle arrest by CDK inhibition does not occur [159]. Nonetheless, checkpoint mutants exhibit a cytologically typical mitotic arrest defect, with elongated spindles in response to blocked replication forks [2,4,160]. Budding yeast cells transmit the checkpoint signal to inhibit progression of mitosis by stabilizing securin^Pds1^, which inhibits the metaphase to anaphase transition, and by stimulating the Bub2/Bfa1 GAP complex which inhibits the mitotic exit network [22,161,162]. Microtubule elongation is also blocked [163].

### 4.2. Essential Function for Cell Viability—dNTP Pool Regulation and Fork Maintenance?

Intriguingly, Mec1 and Rad53 and their functional homologs ATR^Mec1^ and CHK1^Chk1^ are essential for cell proliferation, but this is not due to their checkpoint function [164,165,166,167,168,169]. In *S. cerevisiae*, the lethality of *mec1* or *rad53* deletions can be bypassed by up-regulating the dNTP pool with another mutation [164,165]. In *S. pombe*, on the other hand, neither Rad3^Mec1^ nor Cds1^Rad53^ are encoded by essential genes, raising the question of whether the critical dNTP regulating function of these checkpoint kinases is conserved. In mammalian cells NTP pool control through c-Myc appears to be a determinant of dNTP levels [170].

In *S. cerevisiae* dNTP levels increase 8-fold in response to DNA damage, an increase that facilitates cell survival, even as it increases the mutation rate [171]. Given the pleotropic effects of dNTPs, it is reasonable that cells have multiple pathways that regulate dNTP concentrations, one of which is through the Dun1 kinase, a target of Rad53 [172]. Dun1 phosphorylates Sml1 and Dif1, which inhibit ribonucleotide reductase (RNR), priming them for degradation [173,174]. Dun1 also induces RNR gene transcription by inhibiting the transcriptional repressor Crt1 [175]. Given that the failure to regulate dNTPs is lethal, these pathways are obviously very important in budding yeast.

A recent study from the Longhese laboratory has revealed that the essential functions of Mec1 and Rad53 can also be bypassed by lowering the activity of Cdc28, the budding yeast CDK [176]. Similarly, a delayed entry into S or M phase provoked by lowered levels of G1- or M-phase cyclins, improves the survival of *mec1Δ* or *rad53Δ* cells on low doses of HU. This suggests that either extending G1, prior to S phase entry, or a reduction in the number of active replication forks, compensates for the lethal effects of checkpoint kinase ablation. Most likely, this suppression is explained by the fact that cells have sufficient time both to generate dNTPs and to complete replication. Surprisingly, lowered rates of microtubule elongation provoked by *cin8* mutation, also suppresses the lethality of *mec1Δ* or *rad53Δ* cells. Both survival on HU and the ensuing completion of replication improve in the *cin8* mutant, suggesting that centromere segregation by a premature mitotic spindle, is another lethal consequence of *mec1* or *rad53* ablation. Similarly, the inhibition of microtubule elongation through nocodazole diminishes Rad52 repair foci, which are induced in cells bearing the temperature-sensitive *mec1*-*14* mutation at elevated temperatures in S phase [176]. Together, these data suggest that checkpoint kinases also coordinate the completion of replication with microtubule elongation, in line with the original concept of checkpoints: that is, to preserve the order of cell-cycle events. We note, however, that the experiments in the cited study used either no or low dose HU. Indeed, after treatment with high doses of HU or MMS, the essential function of the replication checkpoint was shown to be its ability to facilitate the restart of replication forks once the lesions have been removed [34,164]. This may act by preventing the accumulation of aberrant DNA structures and/or fork collapse [32,148,149,150] (see Section 4.6).

Mec1 and ATR^Mec1^ are known to prevent chromosome breakage at fragile sites where replication forks frequently slow down, even in the absence of exogenous damage [31,177]. Given the fact that ATR^Mec1^ is an essential protein in mammalian cells, and given that there are many more obstacles that impair fork progression in higher eukaryotes, it may well be that overcoming intrinsic replication stress is the essential role for ATR^Mec1^/CHK1^Chk1^ in higher organisms. 

### 4.3. Replication Origin Control

DNA replication is initiated by a series of steps that proceed in a sequential manner. In the first step, known as licensing, the pre-replicative complex (pre-RC) is loaded onto DNA at the origins of replication in G1 phase, when CDK activity is low. The pre-RC consists of ORC, Cdc6, Cdt1, and Mcm2-7. In the second step, the essential helicase components Cdc45 and GINS, together with DNA polymerases, are brought onto pre-RC by the bridging factors Sld3-Sld7 and Dpb11-Sld2 in a CDK- and DDK- (Dbf4 dependent kinase, CDC7^Cdc7^-DBF4^Dbf4^) dependent manner [178,179]. Finally, Mcm10 functions in the unwinding step, together with the CMG helicase complex (Cdc45-MCM-GINS), thus initiating DNA replication [180,181,182].

In eukaryotes, there are multiple origins of DNA replication (in *S. cerevisiae* ~500 in a haploid genome), and those initiation events are regulated temporally [183,184]. In budding yeast cells, several factors essential for the initiation of DNA replication are limiting, and those factors appear to be recycled for later initiation events [185,186]. Recent studies have shown that Rad53 targets and inactivates two of the limiting replication factors, Sld2 and Dbf4 (the DDK regulatory subunit). Thus, late-origin firing is suppressed by an activated replication checkpoint [187,188]. Regulation of replication initiation by the checkpoint also occurs in higher eukaryotic cells, although the targets appear to be different [189]. It is interesting to note that in *S. pombe* and mammalian cells, the DDK has been shown to have a positive role in replication checkpoint activation [190,191,192]. DDK also modulates the checkpoint response to facilitate DNA repair and the recovery from checkpoint arrest [193,194] (see Section 4.5).

### 4.4. Transcription Control

Genome-wide gene expression analyses in budding yeast have revealed that hundreds of genes are up- or down-regulated upon treatment with genotoxic reagents that induce stalled replication forks [127,195,196,197]. This transcriptional regulation is controlled by two branches in the replication checkpoint pathway; one directly by Rad53, the other by Dun1 [175,198,199]. Dun1 phosphorylates and inhibits Crt1, which recruits repressors Ssn6 and Tup1 to the promoters of DNA damage response genes. Dun1 thereby up-regulates genes involved in DNA repair and ribonucleotide biosynthesis [175,196]. Two recent studies have revealed that the cell-cycle dependent genes that are transcribed at the G1/S boundary are also induced as a part of the DNA replication and damage response in *S. cerevisiae* [198,199]. Over 200 G1/S genes are regulated by the heteromeric transcription factors SBF (Swi4-Swi6 cell-cycle box (SCB) binding factor) and MBF (*MluI* cell-cycle box (MCB) binding factor) [200]. While SBF activates transcription in G1, MBF down-regulates transcription outside of G1 through the co-repressor Nrm1, thereby restricting the expression of the target genes in late G1 [201,202]. The studies revealed that MBF target genes are up-regulated upon replication stress by inactivation of Nrm1 in a Rad53-dependent Dun1-independent manner [198,199]. This transcriptional regulation is also conserved in *S. pombe*, as the Rad53 homolog Cds1^Rad53^ inhibits Nrm1 and promotes G1/S transcription in response to replication stress [202,203].

### 4.5. Coordinating DNA Repair

It seems obvious that the DNA damage checkpoint should be coupled with the up-regulation of DNA repair, and various forms of damage provoke both a checkpoint response and DNA repair. Since ssDNA coated by RPA initiates both checkpoint activation and the loading of Rad51 for repair by HR, the cell has to carefully coordinate these events, particularly at the replication fork where ssDNA exists constitutively. Importantly, studies in budding and fission yeasts have shown that the replication checkpoint actively suppresses the initiation and processing required for HR [204,205,206]. Rad52 foci are absent in cells treated with HU, even in the presence of DSBs, as long as the replication checkpoint is intact [204,205]. Consistently, the ATR^Mec1^-p53 pathway has been shown to suppress the formation of RAD51^Rad51^ foci in response to HU in mammalian cells [207], although in other cases it has been reported that CHK1^Chk1^ phosphorylates RAD51^Rad51^ and positively regulates HR in response to HU or CPT [208,209]. These discrepancies suggest that the checkpoint regulation of HR is fine-tuned with respect to the type and level of damage.

In addition to the role of 9-1-1 in ATR^Mec1^ activation described above, it has been well documented that 9-1-1 functions in various aspects of DNA repair (see review [210]). Indeed, cells may use the multi-tasking capacity of 9-1-1 to coordinate the checkpoint activation with DNA repair. In both *S. cerevisiae* and *S. pombe*, it has been suggested that 9-1-1 functions in DNA damage tolerance pathways [211,212,213]. The budding yeast 9-1-1 complex also contributes to the resection of DSBs, as *rad24* mutants that impair 9-1-1 loading have reduced ssDNA formation and impaired recruitment of Mec1 at HO-induced breaks [48,214]. Finally, human 9-1-1 physically interacts with factors involved in base excision repair (BER), such as MYH, Polβ, TDG, Fen1, and DNA ligase I, and stimulates their enzymatic activities [215,216,217,218,219,220].

How then is 9-1-1 function regulated? Recent studies have indicated that post-transcription modification of 9-1-1 is crucial for its regulation. *S. pombe* Rad9^Ddc1^, a component of checkpoint clamp, is phosphorylated at multiple sites by Rad3^Mec1^, and two phosphoacceptor sites in the C-terminal tail that promote Rad9^Ddc1^-Cut5^Dpb11^ interaction are required for Chk1^Chk1^ activation [53] (see Section 2.1). Interestingly, Rad3^Mec1^-dependent phosphorylation at T225 on Rad9^Ddc1^ has been shown to facilitate the interaction with Mms2^Mms2^-Ubc13^Ubc13^, a ubiquitin-conjugating enzyme, to promote error-free repair [221]. Finally, a study in human cells has indicated that RAD18^Rad18^ facilitates RAD9^Ddc1^ recruitment at IR-induced damage through an unknown mechanism, although this mode of RAD9^Ddc1^ recruitment has little impact on checkpoint activation: no loss of CHK1^Chk1^ or CHK2^Rad53^ activation was scored in cells depleted for RAD18^Rad18^ [222].

As mentioned above, Dbf4-Cdc7 also modulates checkpoint activity [190,191,192,194]. A recent study by Furuya *et al.* has shown that the *S. pombe* DDK phosphorylates Rad9^Ddc1^ [193], thereby reducing interaction between Rad9^Ddc1^ and RPA and releasing 9-1-1 from chromatin. This phosphorylation appears to be important for the repair of CPT-induced lesions, as the number of Rad22^Rad52^ repair foci increase in DDK phospho-acceptor site mutants [193]. Together, the above studies argue compelling that 9-1-1 functions at the interface of repair and checkpoint pathways.

### 4.6. Replication Fork Stability

As briefly discussed in Section 4.2, maintenance of replication fork integrity is the crucial function of the replication checkpoint in response to replication stress (Figure 3). Control of the cell cycle, transcription, and origin firing, while important, are non-essential events based on the following considerations: (1) Blocking the transition through M phase by nocodazole is not sufficient to rescue the lethality of high doses of MMS or HU in *rad53* or *mec1* mutant cells [12,164]; (2) De novo protein synthesis does not contribute to cell viability, nor is it required for the resumption of replication forks after HU treatment [34]; and (3) HU treatment of cells that cannot suppress late-origin firing due to phospho-site mutations in both Dbf4 and Sld3, is not lethal [187,188]. On the other hand, checkpoint mutants fail to resume DNA replication after transient exposure to HU or MMS, and replisome components are not detected by chromatin immunoprecipitation (ChIP) at early origins on HU in *mec1* mutants [32,34,148,150]. Finally, upon fork stalling, checkpoint mutants show aberrant DNA structures, such as the formation of reversed forks (so-called chicken-foot structures) and an accumulation of ssDNA [33,149]. These observations led to the notion that the replication checkpoint maintains the stable association of replication polymerases at stalled forks, and prevents the formation of pathological fold-back structures in face of replication stress. 

An important aspect of the replication checkpoint is the fact that the roles of ATR^Mec1^ and the effector kinase, CHK1^Rad53^, are not equivalent, particularly with respect to fork recovery. This is well-documented, yet often overlooked. In *S. cerevisiae* it has been shown that the function of Rad53 that is essential for viability on MMS is largely rescued by deletion of the exonuclease, *EXO1* [223]. This suggests that the down-regulation of Exo1 at arrested forks is a major function of Rad53 [223]. Interestingly, however, an *EXO1* deletion does not suppress *mec1Δ* lethality, indicating that Mec1 has functions that are crucial for cell survival on MMS, other than the activation of Rad53 [223]. Indeed, when forks are arrested by high concentrations of HU, quantitative ChIP assays showed that leading and lagging DNA polymerases are displaced in *mec1* mutant cells, although their association is intact in the absence of Rad53 [32,148,150]. Similarly, in *mec1* mutants Cdc45 becomes undetectable at early origins [27,32]. In *rad53* mutants, on the other hand, ChIP signals for DNA polymerases stay high, yet the distribution of the MCM helicase is altered, underscoring again the distinct roles played by Mec1 and Rad53 at stalled forks [32,148]. The exact mechanisms are unclear, yet it appears that Mec1 activity keeps replication polymerases engaged in the presence of HU, while Rad53 acts primarily through the MCM helicase to ensure replication restart [32,148].

Whereas the outcome of checkpoint activation is clear, it is not clear exactly what happens to the stalled replisome complex in the absence of a functional replication checkpoint. ChIP data led to the conclusion that the replication checkpoint stabilizes the replisome and preserves its integrity (Figure 3C). Recent biochemical approaches, on the other hand, have shown that intact replisome complexes can be recovered by Sld5-(GINS) pull-down even in *mec1* and *rad53* checkpoint mutants arrested with HU. The replisome complex, even though it is not functionally engaged, may stay chromatin-associated in both checkpoint-proficient and -deficient cells [224]. ChIP coupled with deep sequencing suggests that the replisome moves away from the last site of DNA synthesis in an uncoordinated manner in checkpoint mutants, possibly still unwinding double-stranded DNA by the helicase, but not incorporating dNTPs (Figure 3D). This random sliding of the replisome could explain the apparent “loss” of polymerases from early initiating sites, as well as the accumulation of ssDNA observed by ChIP and electron microscopy analyses [27,32,33,150,224]. This movement, however, was only detected at the very earliest origins [224], did not correlate with the incorporation of nucleotides [27,225], and could not account for the majority of forks where both polymerase and helicase seem to persist even in checkpoint mutants [224]. One model that could reconcile these results suggests that the replication checkpoint keeps the replisome engaged at sites of stalled forks in an as yet undefined way, rather than simply tethering the replisome factors together. Further work is needed to address the molecular mechanisms of how the replication checkpoint facilitates the restart of stalled forks and recovery from replication stress.

Several studies have indicated that the checkpoint regulates the action of nucleases and helicases at stalled forks (Figure 3E,F). Indeed, in *S. cerevisiae* the exonuclease Exo1 is modified and inhibited by the DNA damage checkpoint [223,226], and deletion of *EXO1* has been shown to suppress the accumulation of ssDNA in checkpoint-deficient cells on HU [227]. Similarly, human EXO1 undergoes ubiquitin-mediated degradation on HU, in a manner partly dependent on phosphorylation by ATR^Mec1^ [228]. Recently it has also been shown that the nuclease/helicase Dna2 is a target of the replication checkpoint, and that phosphorylation facilitates its association to DNA following exposure to HU [229]. Dna2 nuclease activity is thought to prevent ssDNA formation by cleaving off ssDNA tails that arise from stalled fork regression [229]. A structure-specific nuclease Mus81-Eme1 is also a target of Cds1^Rad53^ in *S. pombe*, and is released from chromatin upon fork stalling [230]. Finally, as mentioned above, the Sgs1 helicase is also a target of Mec1, and its helicase activity has an important role in replication fork stability [32,101,103,148]. Specifically, when *sgs1*Δ is combined with either *mrc1*Δ or *mec1*-*100*, an S phase-specific *mec1* mutant [112], then total fork collapse occurs in the presence of HU, and neither DNA polymerases nor RPA can be detected at the collapsed fork by ChIP analysis [32,101,103,148]. This results in a dramatic increase in gross chromosomal rearrangements and high levels of cell lethality. The mammalian RecQ helicases BLM^Sgs1^ and WRN^Sgs1^ are also phosphorylated by ATR^Mec1^ and ATM^Tel1^ [231,232], and are also implicated in replication fork recovery after stress [231,233,234]. Whereas Sgs1 helicase does help transduce the checkpoint signal by activating Rad53, the prevention of fork collapse requires its helicase activity, and not simply its ability to bind Rad53, but rather depends on the Sgs1 helicase activity [32,101,103,148]. Together, these data suggest that ATR^Mec1^ and CHK1^Rad53^ target distinct sets of nucleases and helicases to suppress the formation of pathological fork structures that impair replication fork resumption after removal of the damage.

A number of studies has been carried out to identify the targets of the replication and DNA damage checkpoints, and to determine their relevance for the checkpoint response. In Table 3, we list the replication fork associated factors in addition to those mentioned above, that are phosphorylated upon checkpoint activation [235,236,237,238,239,240,241]. While phosphoproteomic studies have identified many, only a few checkpoint targets have been elucidated in depth. For example, RPA1^Rfa1^, the large subunit of RPA, is crucial for ATR^Mec1^ recruitment at the site of damage and is a documented Mec1 target [242], yet the function of its phosphorylation remains unclear. In human cells, the second RPA subunit, RPA32^Rfa2^, is phosphorylated by ATR^Mec1^ and by another PIKK, DNA-PK, upon replication stress, and this phosphorylation is required for a robust checkpoint response and for recovery from fork arrest [243]. DNA polymerase α, the lagging strand polymerase, is a checkpoint target [24], and has an important role in the initiation of the replication checkpoint [244]. Interestingly, the gap-filling lagging strand polymerase, DNA polymerase δ, has been shown to be a target of Mec1 in response to MMS [236], although the importance of its phosphorylation in replication fork stability is unclear. The active replicative helicase CMG complex (Cdc45-MCM-GINS) is one of the common targets of the replication and damage checkpoint in different organisms; MCM2^Mcm2^ (primarily a target of ATR^Mec1^ [245,246,247]), MCM3^Mcm3^ (primarily a target of ATM^Tel1^-CHK2^Rad53^ [245,248,249]), MCM4^Mcm4^ (a target of both ATR^Mec1^ and ATM^Tel1^ pathways [249,250,251]), GINS component Psf1 (a Mec1 target [224]), and PSF2^Psf2^ (a CHK2^Rad53^ target [249]) are all phosphorylated upon checkpoint activation. CHK2^Rad53^ phosphorylation of the CMG inhibits its helicase activity [249], whereas phosphorylation of Mcm2 has been shown to recruit Plk1^Cdc5^ (polo-like kinase 1) on chromatin and promote checkpoint recovery [247] (see also Section 5). Finally, analyses in both budding yeast and human cells have identified specific phosphopeptides of the GINS complex and DNA polymerase ε targeted by ATR^Mec1^ and/or ATM^Tel1^, and not by downstream effector kinases CHK1^Rad53^ (see Table 2 [236,237]), consistent with the distinct roles played by ATR^Mec1^ and the downstream effector kinase CHK1^Rad53^. It is of utmost relevance to find out whether, and how, phosphorylation of these factors impacts replication fork integrity and fork restart.

**Table 3:** Replication factors modified by replication and damage checkpoint kinases.

	Function	References
***S. cerevisiae* (targeted by)**		
**Rfa1 (Mec1/Tel1)**	a subunit of RPA	[235,236,252]
**Rfa2 (Mec1/Tel1)**	a subunit of RPA	[236,242]
**Pol1 (Rad53)**	a subunit of DNA polα	[236]
**Pol12 (Rad53)**	a subunit of DNA polα	[24]
**Pol31 (Mec1/Tel1)**	a subunit of DNA polδ	[236]
**Dpb4 (Mec1/Tel1)**	a subunit of DNA polε and ISW2	[235,236]
**Mcm4(Mec1) **	a subunit of MCM	[21]
**Mcm6 (Mec1)**	a subunit of MCM	[21]
**Psf1 (Mec1)**	a subunit of GINS	[224]
**Mrc1 (Mec1, Rad53)**	checkpoint mediator	[25,236]
**Tof1 (Rad53)**	fork protection complex	[235]
**Ctf4 (Rad53)**	polα interactor	[235]
**Dbf4 (Rad53)**	a subunit of DDK	[187,188,236,253]
**Sgs1 (Mec1)**	RecQ helicase	[103]
**Higher eukaryotes**		
**RPA1 (ATR/ATM)**	a subunit of RPA	[237,241]
**POLE (ATR/ATM)**	a subunit of polε	[237]
**POLEE4 (ATR/ATM)**	a subunit of polε	[237]
**POLL (ATR/ATM)**	a subunit of polλ	[237]
**MCM2 (ATR/ATM)**	a subunit of MCM	[237,239,241]
**MCM3 (ATR/ATM)**	a subunit of MCM	[237]
**MCM4 (ATR/ATM, CHK1)**	a subunit of MCM	[237,239,250]
**MCM5 (CHK1)**	a subunit of MCM	[240]
**MCM6 (ATR/ATM)**	a subunit of MCM	[237,239]
**MCM7 (ATR/ATM)**	a subunit of MCM	[237]
**POLDIP3 (ATR/ATM)**	Polδ interacting factor	[237]
**MCM10 (ATR/ATM)**	replication initiation factor	[237]
**HELB (ATR/ATM)**	DNA helicase B	[237]
**RFC1 (ATR/ATM)**	clamp loader	[237,239,241]
**RFC3 (ATR/ATM)**	clamp loader	[237]
**PSF2 (ATR/ATM)**	a subunit of GINS	[237,241,249]
**ORC3 (ATR/ATM)**	a subunit of ORC	[237]
**ORC6 (ATR/ATM)**	a subunit of ORC	[237]
**Higher eukaryotes**		
**DBF4 (ATR/ATM)**	a subunit of DDK	[237]
**Claspin (ATR/ATM)**	checkpoint mediator	[237,241]
**CAF-1B (ATR/ATM)**	histone assembly	[241]
**CTF18 (ATR/ATM)**	POLH interactor	[237,241]
**TopBP1 (ATR/ATM, CHK1)**	initiation and ATR activation	[237,240,241]
**WDHD1 (ATR/ATM)**	Polα interactor	[241]
**BLM (ATR/CHK1)**	RecQ helicase	[241,240]
**FEN1 (CHK1)**	5' flap endonuclease	[240]
**DNA Ligase 1 (CHK1)**	DNA ligase	[239,240]
**TIPIN (ATR/ATM)**	fork protection complex	[237]
**WRN (ATR/ATM)**	RecQ helicase	[232,237]

In addition to direct fork-associated proteins, enzymes that modulate long-range chromatin organization are also shown to be targets of the replication checkpoint. For instance, the Ino80 and Isw2 chromatin remodelers are confirmed targets of the DNA damage checkpoint [236,254]. These ATP-dependent chromatin remodeling complexes are shown to be recruited at the stalled replication forks and to promote the recovery of stalled forks [255,256,257,258]. Loss of Ino80 chromatin remodeling activity results in a poor resumption of stalled forks and an increase in DNA repair response [255,256,258]. This correlates with a proposed action of removing nucleosomes to allow fork progression, although INO80 contains a 5' to 3' DNA helicase activity in its Rvb1 and Rvb2 subunits, which may also be involved in altering fork structure [259]. Another study has suggested a role in checkpoint down-regulation, although how chromatin remodeling reduces a checkpoint response is unknown ([260] see also Section 5.2).

A recent study has proposed that the replication checkpoint releases topological tension generated by a transcribed gene that is tethered to components of the nuclear pore [261]. DNA replication forks frequently pause at transcribed genes, and this pausing is independent of the directionality between replication and transcription [62]. Bermejo *et al.* [261] have determined that mutations in THO, TREX-2, or inner basket nucleoporins enhance the survival of *rad53* mutants on HU, and rescue fork reversal, which occurs in the checkpoint mutants on HU [261]. The replication checkpoint appears to target Mlp1, a nucleoporin, and counteracts its function for gene tethering to the nuclear pore. A phospho-mimicking *mlp1* mutation suppresses *rad53*Δ lethality on HU, suggesting that releasing topological impediments generated by gene gating is one task of the replication checkpoint. However, the interpretation may be rather complicated since loss of Mlp1 also releases and alters the activity of the SUMO-protease Ulp1 which plays a key role in DNA repair [262].

A bioinformatics approach has also indicated that ATR^Mec1^ and ATM^Tel1^ may be involved in regulating a broad range of cellular structures, such as the spindle pole body/centrosome and actin cytoskeleton [263]. It will be interesting to explore whether those less canonical Mec1/Tel1 targets function at replication forks to ensure genome stability.

## 5. Checkpoint Recovery

### 5.1. Protein Phosphatases Down-Regulate the Checkpoint

**Figure 4:**
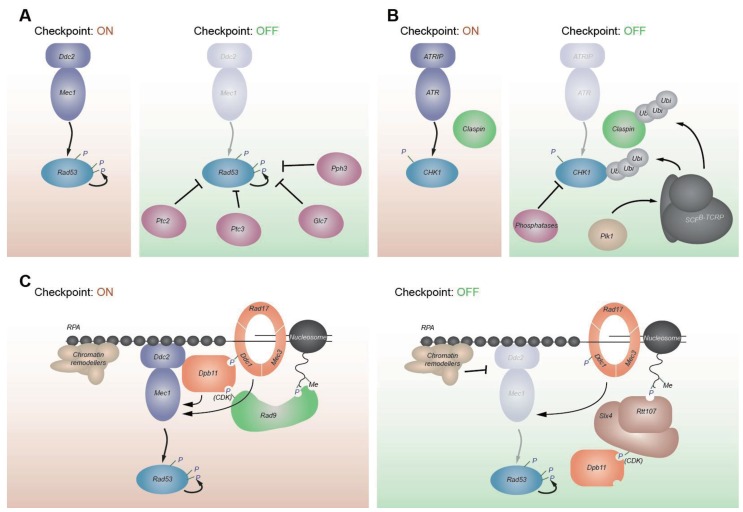
Checkpoint down-regulation. (**A**) The phosphatases Ptc2, Ptc3, Glc7, and Pph3 have been implicated in dephosphorylating Rad53 in *S cerevisae*. (**B**) In the mammalian replication checkpoint, CHK1^Chk1^ activation by ATR^Mec1^ is facilitated through the checkpoint mediator Claspin^Mrc1^. During checkpoint down-regulation, both CHK1^Chk1^ and Claspin^Mrc1^ are targeted for ubiquitin-mediated degradation, which is promoted by Plk1^Cdc5^. In addition, phosphatases lead to CHK1^Chk1^ dephosphorylation. Ubi—Ubiquitin. (**C**) Phosphatase-independent down-regulation in *S.* *cerevisiae*. Left: The Mec1 activator Dpb11 interacts with phosphorylated Rad9. Rad9 is recruited by two chromatin modifications; histone H2A phosphorylation and methylated histone H3, and assists in Rad53 activation. Right: A complex of Slx4 and Rtt107 down-regulates the checkpoint. Slx4 competes with Rad9 for Dpb11 binding, and may sequester Dpb11 away. Rtt107 can interact with phosphorylated H2A, and may compete with Rad9 for the interaction. Chromatin remodelers may also have a role in checkpoint down-regulation.

The kinase cascade of DNA replication and damage checkpoints must be down-regulated to continue the cell cycle once the impediment is removed (Figure 4). This process is known as recovery, and correlates with the disappearance of hyper-phosphorylated Rad53 in *S. cerevisiae*. Recovery is distinct from “adaptation”, which refers to the down-regulation of the checkpoint despite the persistence of unrepaired DNA damage (see review [264]). A reasonable way to counteract the kinase cascade would be through protein phosphatases (see review [265]). In *S. cerevisiae*, PP2C phosphatases Ptc2 and Ptc3, and PP4 protein phosphatase Pph3 have been shown to function in the recovery from HO endonuclease-induced single DSB response [266,267,268]. Rad53 down-regulation requires Pph3 and Ptc2 in response to MMS [269,270] and another PP1 phosphatase, Glc7, in response to HU [271] (Figure 4A). The dephosphorylation of Rad53 is important for the resumption of stalled replication forks after removal of the drugs [270,271]. It has been suggested that the phosphatases non-redundantly target Rad53 in response to different types of damage (DSBs *vs.* MMS) [265]. However, the loss of those phosphatases at the same time impairs cell growth, suggesting that they have overlapping functions that support cell viability [271].

A role for protein phosphatases in checkpoint recovery is conserved in other eukaryotes: PP1 phosphatase Dis2^Glc7^ negatively regulates Chk1^Chk1^ in *S. pombe* [272] and, in metazoans, various phosphatases have been found to function in checkpoint down-regulation (see reviews [273,274]). ATM^Tel1^ is targeted by PP2A and Wip1 (Wild-type p53-induced phosphatase 1) [275,276], as are CHK1^Chk1^ and CHK2^Rad53^ [277,278,279,280,281]. Wip1 activity is cell-cycle regulated by both protein level and phosphorylation, and it has been suggested that Wip1 protein regulation fine-tunes the global DDR response [282].

### 5.2. Phosphatase-Independent Mechanisms of Checkpoint Down-Regulation

In addition to the phosphatase-dependent down-regulation, the ubiquitin proteasome pathway also plays a role in checkpoint recovery in higher organisms (Figure 4B). CHK1 has been shown to be targeted by cullin 1 (CUL1) or cullin 4A (CUL4A) dependent proteolysis [283,284]. Claspin^Mrc1^ is also targeted by SCF^Β-TRCP^ for degradation [285,286,287], and this effect is counteracted by USP7, a deubiquitylating enzyme, called ubiquitin-specific protease 7 [288]. PLK1^Cdc5^ (polo-like kinase 1) promotes Claspin^Mrc1^ degradation by promoting SCF^Β-TRCP^-dependent ubiquitination [285,286,287], and this PLK1^Cdc5^ activity is prompted by Aurora A and Greatwall kinases [289,290]. The role of PLK1^Cdc5^ in checkpoint down-regulation may be conserved, since the *S. cerevisiae* PLK1 homolog, Cdc5, is important for adaptation from an irreparable DSB, although the action of Cdc5 appears to be different [122,291,292,293]. PLK1^Cdc5^ has also been shown to facilitate DNA replication under replication stress. Namely, the *Xenopus* PLK1^Cdc5^, Plx1^Cdc5^, is recruited to chromatin through ATR^Mec1^-dependent phosphorylation of MCM2^Mcm2^ at serine 92 upon fork stalling, and Plx1^Cdc5^ recruitment is important for origin firing near stalled forks [247]. In human cells, it has been shown that PLK1^Cdc5^ phosphorylates ORC2^Orc2^ on serine 188. This phosphorylation promotes DNA replication under replication stress [294]. In summary, checkpoint recovery is promoted by two opposing enzymatic activities; multiple phosphatases directly quench the checkpoint kinases and the effectors, while polo-like kinase promotes the degradation of checkpoint factors and facilitates the initiation of replication to complete S phase.

Srs2 helicase and Sae2 in *S. cerevisiae* appear to facilitate checkpoint recovery by eliminating or reducing the source of the sensor kinase activation. Srs2 restrains homologous recombination by dislodging the Rad51 recombination protein from ssDNA [295,296]. Consistently, *SRS2* deletion impairs the down-regulation of the checkpoint after repair of HO-induced DSBs [297,298]. A recent study has demonstrated that *srs2*Δ cells retain Ddc2- and RPA-focus formation and chromatin association, even when the bulk of the DSB repair has been completed [299]. Yeung and Durocher suggest that Srs2 dismantles Rad51, which results in elimination of ssDNA and a suppression of Mec1 signaling. Sae2 (CtIP in vertebrates) is involved in meiotic and mitotic DSB processing, together with the Mre11-Rad50-Xrs2 (NBS1 in vertebrates) complex [300,301,302,303]. MRN^MRX^ recognizes DSBs very rapidly and recruits ATM^Tel1^ kinase. Several studies have shown that *sae2* mutants show a defect in checkpoint recovery, whereas overexpression of *SAE2* counteracts checkpoint activation [304,305,306]. Deletion of *SAE2* results in an increased level of MRX at DSBs, whereas overexpression of *SAE2* has the opposite effect, suggesting that Sae2 limits MRX retention at the damage site and thereby limits Tel1 checkpoint signaling [304,305]. This Sae2 function may facilitate switching from Tel1/ATM to Mec1/ATR signaling (see Section 6).

Recent studies in *S. cerevisiae* have shed light on yet another mechanism of the checkpoint attenuation that involves a scaffold complex called Slx4-Rtt107 (Figure 4C). The Mec1 activator Dpb11 binds Rad9 and up-regulates Rad53 activation (see Section 2.4) [93], and the Mec1/Tel1 phosphorylation of H2A at Ser129 (H2A-p; γH2AX in mammals) recruits Rad9 to damage sites, also leading to the activation of Rad53 [307]. Intriguingly, the DNA repair scaffold complex Slx4-Rtt107 can also bind Dpb11 in a Mec1-dependent manner [308], and the C-terminal tandem BRCT domain of Rtt107 interacts with H2A-P [309]. In a recent study, the Smolka group argues that the Slx4-Rtt107 complex negatively regulates Rad53 by competing with Rad9 for both Dpb11 and H2A-p, two positive checkpoint regulators [310]. 

Finally, a recent study suggests that both Isw2 and Ino80, confirmed targets of checkpoint kinases, interact with RPA and function to attenuate the checkpoint activity [260]. Molecular mechanisms of the remodeler function in checkpoint recovery are unclear, but a similar competition may exist where Ino80 and Isw2 exclude excess Mec1 from RPA binding.

## 6. Coordination between ATR^Mec1^ and ATM^Tel1^

While ATR^Mec1^ and CHK1^Chk1^ serve distinct in the replication checkpoint, it is also clear that ATR^Mec1^ and ATM^Tel1^ do not have identical roles. ATM^Tel1^ initiates the DSB checkpoint response, while ATR^Mec1^ is the first responder to the replication-associated DNA damage. As mentioned above, MRX/MRN recognizes DSBs and recruits ATM^Tel1^ kinase. However, there are also a range of overlapping functions between ATM^Tel1^ and ATR^Mec1^. At DSBs, budding yeast Tel1 phosphorylates Mre11, Xrs2, and Sae2. Sae2 activates MRX nuclease activity, which initiates 5'–3' end resection [311]. Extended ssDNA formation is followed by Exo1, Sgs1-Top3-Rmi1, and Dna2 helicase/nuclease activity, with the aid of Fun30 (SMARCAD1), a recently identified chromatin remodeler [311,312,313,314]. Extended RPA-coated ssDNA leads to the recruitment and subsequent activation of ATR^Mec1^-ATRIP^Ddc2^ [39] (see Figure 1). Recently, Peterson *et al.* have shown that ATR^Mec1^ in mammals also phosphorylates CtIP^Sae2^, and this phosphorylation stimulates CtIP^Sae2^ chromatin interaction and end-resection [315]. Thus, checkpoint signaling after DSB induction involves both the ATR^Mec1^ and ATM^Tel1^ pathways. A recent biochemical analysis has suggested a mechanism for a consecutive switch from ATM^Tel1^ to ATR^Mec1^ through swapping the recruiter MRN^MRX^ for RPA at the DSB damage site during end-resection [40]. We note that in higher eukaryotes another PIKK, DNA-PK, also participates in the signaling of the DSB damage response [316].

Although ATR^Mec1^-ATRIP^Ddc2^ is the major regulator of the replication-associated checkpoint, the vulnerable nature of replication forks can generate DSBs through fork collapse (Figure 1), which then might require ATM^Tel1^ activation to protect the break and facilitate fork restart. In a *Xenopus* replication system, both ATM^Tel1^ and ATR^Mec1^ appear to play an important role in preventing DSB formation during replication [317]. In the absence of ATM^Tel1^ and ATR^Mec1^, DSBs accumulate and DNA polymerase ε is displaced from CPT- or mitomycin C-damaged chromatin. It has also been demonstrated that MRN^MRX^ is redistributed to the restarting forks, in a manner dependent on ATM^Tel1^ and ATR^Mec1^. This appears to be important for preventing persistent DSB formation [317]. Consistently, Doksani *et al.* have shown that in the absence of Tel1 or MRX function, abnormal DNA intermediates accumulate when the replication fork encounters an induced DSB. They suggest that the MRX-Tel1 pathway prevents the formation of cruciform structures at the fork-DSB junction [318], a pathway that may depend on crosstalk between ATR^Mec1^ and ATM^Tel1^. 

The mechanism of ATR-ATM crosstalk is somewhat lesion specific. For instance, ATR^Mec1^ phosphorylates ATM^Tel1^ at serine 1981, which promotes the dissociation of inactive ATM^Tel1^ dimers into active monomers, in response to HU or UV. This results in CHK2^Rad53^ activation and a robust G2/M arrest [319]. In contrast, in the presence of IR-induced damage, ATM^Tel1^ auto-phosphorylates this same residue, resulting in self-activation [108,319]. Screens for novel *tel1* mutations in budding yeast has shown that there are overlapping functions between Mec1 and Tel1 with respect to stalled forks, and that some *tel1* gain-of-function mutants can even compensate for Mec1 functions. These dominant *TEL1-hy* mutants suppress *mec1Δ* deficiency in respect to Rad53 activation and cell survival to different extents, in response not only to DSBs but also to UV, MMS, and HU in S phase [320]. Some Tel1-hy proteins have higher catalytic activity than the wild-type Tel1, yet others show equivalent or even lower activity, suggesting that changes in specificity can also result in suppression. In addition, checkpoint restoration by *TEL1-hy* mutants in response to DSBs requires Xrs2 interaction, suggesting that Tel1-hy recruitment to damage through MRX is still crucial for the suppression phenotype [320].

In agreement with the above observations, MRX has been shown to have a role in replication checkpoint signaling, in parallel to its well-characterized function in the DSB response [79,321]. Indeed, budding yeast *mre11* mutants show reduced Rad53 activation in response to HU [321]. In mammals, as well, MRN^MRX^ has been shown to function upstream of ATR^Mec1^ in response to UV and HU, and to interact with ATR^Mec1^-ATRIP^Ddc2^ through the FHA and BRCT domains of Nbs1^Xrs2^ [79]. These data would suggest that MRN^MRX^ functions as a regulator for both ATM^Tel1^ and ATR^Mec1^. Recently, MRN^MRX^ has also been found to assist in TopBP1^Dpb11^ recruitment to ssDNA-dsDNA junctions, explaining how this complex might help activate ATR^Mec1^ in *Xenopus* egg extracts [89,322]. It is proposed that MRN^MRX^ recruits TopBP1^Dpb11^, after which the interaction between 9-1-1 and TopBP1^Dpb11^ subsequently exposes the ATR^Mec1^ activation domain (AAD) of TopBP1^Dpb11^ to fully activate ATR^Mec1^ [89]. On the other hand, the Mre11 nuclease activity has also been implicated in checkpoint activation, possibly by generating ssDNA [322]. In budding yeast MRX is also recruited to stalled replication forks, where it contributes to the maintenance of stalled polymerases [323]. Another study by the same group has demonstrated that MRX has an important role in cohesin association behind the fork in response to HU, which is necessary for fork restart [323,324]. Whereas the full molecular details of how MRX contributes to fork stability and cohesin recruitment remain to be clarified, current results suggest that it may be its structural properties, rather than its nuclease activity, that are crucial [323,324]. In mammalian cells cohesin is also important for CHK2^Rad53^ activation in the intra-S and the G2/M damage checkpoints, and this role is independent of cohesin’s role in sister chromatid cohesion [325]. In *S. pombe*, the N-terminus of the MRN component Nbs1^Xrs2^ is required for the Rad3^Mec1^-Rad26^Ddc2^-dependent telomere recruitment of Tel1, while the well-studied Tel1-binding C-terminal Nbs1^Xrs2^ domain is not needed [326]. These observations further emphasize the significant cooperation that exists between ATR^Mec1^ and ATM^Tel1^, and the important role MRN^MRX^ plays in coordinating their interplay. 

## 7. Perspectives

The replication checkpoint is a highly conserved surveillance mechanism. Here we have summarized both past and recent insights into the mechanistic aspects of the DNA damage checkpoint response. We focused on the replication checkpoint because of the important role played by the DNA damage response to DNA replication stress in the early stages of human tumorigenesis. It has been amply demonstrated that the DNA damage response, monitored as γH2AX or 53BP1^Rad9^ foci formation, or CHK2^Rad53^ phosphorylation is constitutively activated in precancerous lesions, even in the absence of exogenous damage [327,328]. It has been suggested that the DNA damage checkpoint is an important barrier for suppressing cancer development, acting at least in part through p53-dependent senescence [7,327,328,329,330]. The persistent DNA damage response that is detected in precancerous lesions or in hyperplastic tissues results from inappropriate initiation of DNA replication and the ensuing DNA replication stress [329,330]. We note that p53 activation in response to DSBs is primarily mediated by ATM^Tel1^ and CHK2^Rad53^ [331], whereas the apoptosis induced by replication stress is independent of p53 [332,333,334]. Thus, p53-deficient cancer cells may rely on ATR^Mec1^-CHK1^Chk1^ for their survival during replication stress or fork-associated DNA damage. This type of damage is often enhanced by drugs used as chemotherapeutics, namely alkylating chemicals, cis-platin, aphdicolin, or HU. Indeed, ATR^Mec1^ inhibition has been shown to exacerbate the toxicity of replication stress in p53-deficient cells [335], and CHK1^Chk1^ inhibition sensitizes p53 mutants to ionizing radiation [336]. Thus, the ATR^Mec1^-CHK1^Chk1^ replication checkpoint pathway has great potential for improving cancer chemotherapy strategies. In conclusion, continued research into the replication checkpoint is needed to be able to design novel and more effective chemotherapeutics and combined therapies for human cancer. A deeper understanding of how the replication checkpoint maintains replication fork integrity may enable us to better exploit this natural “Achilles heel” of cancer cells for more effective therapies.
